# Air Pollution-Associated Rhinitis: Exploring the Preventive Role of Nutritional Supplements Against Particulate Matter-Induced Inflammation

**DOI:** 10.3390/nu17050829

**Published:** 2025-02-27

**Authors:** Shih-Wei Chen, Stella Chin-Shaw Tsai, Kuang-Hsi Chang, Kai-Cheng Chuang, Muhammad Sufian, Hueng-Chuen Fan, Chuan-Mu Chen

**Affiliations:** 1 Department of Otolaryngology, Tungs’ Taichung MetroHarbor Hospital, Taichung 435, Taiwan; eddielodz@gmail.com (S.-W.C.); tsaistella111@gmail.com (S.C.-S.T.); 2 Ph.D. Program in Translational Medicine, Department of Life Sciences, National Chung Hsing University, Taichung 402, Taiwan; dpachuang@gmail.com (K.-C.C.); sufianzoologist@gmail.com (M.S.); 3 Rong Hsing Research Center for Translational Medicine, College of Life Sciences, National Chung Hsing University, Taichung 402, Taiwan; 4 Department of Medical Research, Tungs’ Taichung Metroharbor Hospital, Taichung 435, Taiwan; kuanghsichang@gmail.com; 5 Center for General Education, China Medical University, Taichung 404, Taiwan; 6 General Education Center, Jen-Teh Junior College of Medicine, Nursing and Management, Miaoli 356, Taiwan; 7 Institute of Molecular Biology and Biotechnology (IMBB), The University of Lahore, Lahore 54000, Pakistan; 8 Department of Pediatrics, Tungs’ Taichung Metroharbor Hospital, Taichung 435, Taiwan; 9 Department of Pediatrics, Tri-Service General Hospital, National Defense Medical Center, Taipei 114, Taiwan; 10 The iEGG and Animal Biotechnology Research Center, National Chung Hsing University, Taichung 402, Taiwan; 11 Center for General Educational, National Quemoy University, Kinmen 892, Taiwan

**Keywords:** air pollution, rhinitis, particulate matter, PM4.0, PM2.5, inflammation

## Abstract

Air pollution, particularly particulate matter (PM), poses a significant health risk worldwide, with rhinitis emerging as a prevalent respiratory condition. This review explores the association between air pollution and rhinitis, focusing on PM-induced inflammation and the potential preventive role of nutritional supplements. A comprehensive literature search was conducted using the PubMed and Scopus databases, covering studies from inception to 2024 that investigated air pollution, rhinitis, and nutritional interventions. This review synthesizes evidence linking PM exposure to increased prevalence and exacerbation of rhinitis through various inflammatory mechanisms. We further examine the potential of nutritional supplements, including kefir peptides, lactoferrin, vitamin D, polyunsaturated fatty acids, and probiotics, in mitigating PM-induced inflammation and rhinitis symptoms. However, the evidence regarding the role of these supplements in modulating immune responses and reducing inflammation related to PM-induced rhinitis is limited. This review highlights the potential efficacy of nutritional interventions in preventing and managing air pollution-associated rhinitis, offering a complementary approach to environmental regulations in addressing this public health challenge.

## 1. Introduction

Air pollution has emerged as a significant global health concern, with particulate matter (PM) being one of its most harmful components. In Taiwan, a densely populated island nation with rapid industrialization, air pollution poses a particularly pressing challenge to public health [1]. The primary sources of air pollution include industrial emissions, transport emissions, coal combustion, and waste incineration [1]. In recent years, the awareness of the long-term health effects of air pollution exposure has increased. Studies indicate that approximately 3.3 million people worldwide die prematurely due to outdoor air pollution exposure annually, with Asian populations being disproportionately affected [2].

Among the myriad health issues associated with air pollution, respiratory diseases have garnered significant attention. Rhinitis, characterized by inflammation and swelling of the nasal mucosa, has become one of the most prevalent chronic illnesses worldwide [3]. In Taiwan, the prevalence of allergic rhinitis (AR) is particularly high at 25%, while non-allergic rhinitis (NAR) affects 20% of the population [4,5,6]. The increasing prevalence of rhinitis, especially in developed and developing countries, has prompted researchers to investigate potential risk factors, with environmental factors, including air pollution, emerging as significant contributors [7,8].

Particulate matter, especially fine (PM2.5) and coarse (PM10) particles, has been strongly linked to the development and exacerbation of rhinitis. These particles can act as irritants and adjuvants, enhancing allergic responses and triggering inflammation in the nasal mucosa [9]. PM exposure can increase the permeability of the nasal epithelium, facilitate allergen penetration, and enhance the activation of dendritic cells and T cells [10]. Moreover, PM can increase the production of pro-inflammatory cytokines and enhance IgE production, further contributing to the pathogenesis of rhinitis [10].

In addition, airborne pollen allergens have been a concern for more than a century, with research indicating a link between pollen exposure and allergic rhinitis in individuals with atopic conditions [11,12,13]. The chemical interactions between pollen and air pollutants can modify the protein content within the pollen. When the pollen ruptures, these proteins, along with sub-pollen particles and pollen-associated lipid mediators, are released. Such rupturing events can lead to coexposure, potentially intensifying allergic reactions [14].

The complex relationship between air pollution and rhinitis in Taiwan presents a unique public health challenge. While efforts to reduce air pollution are crucial, there is also a growing interest in exploring preventive strategies to mitigate its harmful effects on respiratory health. In this context, nutritional supplements have emerged as a promising avenue for research due to their potential immunomodulatory and anti-inflammatory properties [15]. This review aims to explore the association between air pollution and rhinitis in Taiwan, with a particular focus on the role of particulate matter in inducing inflammation. Furthermore, we will examine the potential preventive role of nutritional supplements in mitigating the harmful effects of air pollution on nasal health. By synthesizing the current evidence, we hope to provide insights into potential strategies for reducing the burden of air pollution-associated rhinitis in Taiwan and similar environmental contexts.

## 2. Materials and Methods

### 2.1. Literature Search Strategy

A comprehensive literature search was conducted to identify relevant studies on air pollution-associated rhinitis in Taiwan and the potential preventive role of nutritional supplements against particulate matter-induced inflammation. Both the PubMed and Scopus electronic databases were searched. The last search was conducted on 20 September 2024, covering articles published from inception to this date.

### 2.2. Search Terms

The search strategy employed a combination of Medical Subject Heading (MeSH) terms and free-text keywords. The following search terms were used in various combinations:

- Air pollution-related terms: “air pollution”, “particulate matter”, “PM2.5”, “PM10”, “air quality”, “environmental pollutants”
- Rhinitis-related terms: “rhinitis”, “allergic rhinitis”, “non-allergic rhinitis”, “chronic rhinosinusitis”, “nasal inflammation”
- Nutritional supplement-related terms: “nutritional supplements”, “dietary supplements”, “nutraceuticals”, “kefir”, “lactoferrin”, “vitamin D”, “polyunsaturated fatty acids”, “omega-3”, “probiotics”
- Inflammation-related terms: “inflammation”, “inflammatory response”, “cytokines”, “oxidative stress”, “immune modulation”

An example of a search string used in PubMed is as follows:

- ((air pollution [MeSH Terms]) OR (particulate matter) OR (PM2.5) OR (PM10)) AND ((rhinitis[MeSH Terms]) OR (allergic rhinitis) OR (chronic rhinosinusitis)) AND (Taiwan[MeSH Terms]) AND ((nutritional supplements [MeSH Terms]) OR (kefir) OR (lactoferrin) OR (vitamin D) OR (polyunsaturated fatty acids) OR (probiotics))

### 2.3. Inclusion and Exclusion Criteria

This review included original research articles, systematic reviews, meta-analyses, studies conducted in Taiwan or on Taiwanese populations, studies investigating the association between air pollution and rhinitis or respiratory pathology, and studies examining the effects of nutritional supplements on air pollution-induced inflammation or rhinitis. Full-text articles available in English or Chinese were considered. We excluded case reports, conference abstracts, opinion pieces, and animal studies without clear relevance to human health.

### 2.4. Data Synthesis

Due to the expected heterogeneity in the study designs, populations, and outcome measures, a narrative synthesis approach was adopted. The findings were summarized and presented thematically, focusing on the association between air pollution and rhinitis in Taiwan and the potential preventive role of various nutritional supplements against particulate matter-induced inflammation.

## 3. Results and Discussion

### 3.1. Health Impacts of Air Pollution

Due to Taiwan’s unique location, its air quality is frequently affected by dust and anthropogenic aerosols that originate from the Asian continent. Previous evidence indicated that long-range transport influenced fine-sized PM and polycyclic aromatic hydrocarbons (PAHs) rather than coarse-sized ones in Taiwan [16]. The study also found the concentration of PM2.5-bound toxic metals and PAHs increased by 90% due to long-range transport [16]. The toxicity of PM increases when metals, sulfates, PAHs, and dioxins attach to its surface [17,18,19,20]. In Taiwan, industrial emissions are the primary source of air pollution, followed by transport emissions, coal combustion, fossil fuel combustion, and waste incineration [1]. In recent years, there has been a growing awareness of the health effects of long-term exposure to air pollution. A study found that around 3.3 million people worldwide die prematurely due to outdoor air pollution exposure every year, especially in Asian populations [2]. A large body of evidence has demonstrated the significant associations between air pollution exposure and various diseases such as cardiovascular disease, cerebrovascular disease, autoimmune diseases, degenerative skeletal disorders, hearing loss, malignant tumors, and child development [21,22,23,24,25,26,27,28]. Long-term air pollution exposure may also cause tissue-specific inflammation [29,30,31].

### 3.2. Particulate Matter and Its Impact on Respiratory Health

One of the primary components of air pollution is PM, which contains a wide range of hazardous substances such as carbon aerosols, polycyclic aromatic hydrocarbons (PAHs), benzoquinone, and heavy metals [32]. PM can further be divided based on diameter into coarse particles (2.5–10 μm), fine particles (<2.5 μm), and ultrafine particles (<0.1 μm). PM of all sizes can affect our organs to varying extents [33].

The seminal publication by the International Commission on Radiological Protection (ICRP) in 1994 catalyzed research into PM transmission and deposition within the human respiratory tract [34]. Particle deposition is influenced by a multitude of factors, including particle size, specific regions of the upper respiratory tract, and variations in breathing and ventilation patterns. For instance, ultrafine particles smaller than 0.01 μm as well as coarse particles with sizes ranging 2.5–10 μm are deposited in the nasal cavity, pharyngeal, and laryngeal regions [34]. In addition, ultrafine particles are deposited deeper inside the lungs because of their small size, penetrating the epithelial cells, entering the circulatory system, and even crossing into cells and affecting intracellular stability [35,36].

PM has been linked to the inflammation and pathology of the respiratory tract. PM2.5 may enter the bronchial tree and ultimately deposit in the alveoli and terminal bronchioles, causing pulmonary inflammation [37]. High PM2.5 concentrations can trigger the release of tumor necrosis factor-α (TNF-α) and Th2-mediated inflammatory and allergy factors such as interleukin (IL)-4, IL-5, and IL-10 [10]. Sompornrattanaphan et al. correlated PM to allergic respiratory diseases, including AR [38]. On the other hand, PM4.0 is mainly found in the tracheal, nasal cavity, and pharyngeal regions. A longitudinal study conducted by a Taipei hospital assessed the respiratory impacts of indoor respirable dust (PM4.0) in 50 managers. The results revealed that PM4.0 concentration was positively correlated with the daily variation in forced expiratory volume (FEV1); for every increase of 1 mg/m^3^ in PM4.0, the daily variation in FEV1 increased to 0.02. This shows that indoor PM4.0 in hospitals is a risk factor for adverse respiratory effects [39].

### 3.3. The Association Between Particulate Matter and Rhinitis

Rhinitis, which is characterized by inflammation and swelling of the nasal mucosa, has become one of the most prevalent chronic illnesses worldwide. In Taiwan, the prevalence of AR is particularly high at 25%, while NAR affects 20% of the population [4,5,6]. The increasing prevalence of rhinitis, especially in developed and developing countries, has prompted researchers to investigate potential risk factors, with environmental factors, including air pollution, emerging as significant contributors [40,41,42,43].

PM has garnered considerable attention in relation to rhinitis. The potential mechanisms by which PM contributes to rhinitis involve complex interactions with the nasal mucosa and immune system. In the context of AR, PM can act as an irritant and an adjuvant, enhancing the allergic response. When inhaled allergens bind with receptors such as toll-like receptors (TLRs) on epithelial cells in the nasal cavity, they can trigger allergic immune responses [44]. Of particular interest is TLR-4, which is widely expressed on cell surfaces and, when activated, initiates pro-inflammatory responses. TLR-4 is a crucial receptor that mediates the convergence of both infectious stimuli, like pathogen-associated molecular patterns (PAMPs) from bacteria, viruses, fungi, and plants, as well as noninfectious signals, such as ischemia/reperfusion (I/R) injury and neurodegenerative diseases, leading to the activation of pro-inflammatory responses [45]. TLR-4-mediated inflammatory immune responses can also be induced by PM [46]. PM exposure can potentially amplify this process by increasing the permeability of the nasal epithelium, facilitating allergen penetration, and enhancing the activation of dendritic cells and T cells [47,48]. It can also increase nasal inflammation and enhance IgE production [44].

A comprehensive meta-analysis, which pooled data from six birth cohorts, reported a significant correlation between exposure to PM2.5 at birth and the subsequent diagnosis of AR in children aged 7–8 years [49]. The study found that for every 5 µg/m^3^ increase in PM2.5 concentration, the odds of AR diagnosis increased by 37% (OR: 1.37, 95% CI: 1.01–1.86). Further supporting this association, a focused analysis was conducted on a subset of children from a large Dutch birth cohort study comprising 3863 participants. This subgroup consisted of children who had maintained the same residence throughout their first eight years of life, thereby providing a more controlled environment for assessing long-term PM2.5 exposure effects. The researchers identified a notable link between PM2.5 exposure and the occurrence of AR in this population [50].

Chronic rhinosinusitis (CRS), another form of rhinitis, has also been associated with PM exposure. Its pathogenesis remains unclear, but involves pro-chronic inflammatory factors including chronic allergies, nasal polyps, and environmental stimulants. A South Korean nationwide population study demonstrated a positive correlation between PM10 concentration and the risk of CRS [51]. A Chinese time-series study provided further insights into the relationship between air pollutants and CRS. After adjusting the exposure–response curves for six air pollutants, the study found that PM2.5 and PM10 concentrations showed a sharp increase in CRS risk at low concentrations, followed by a gradual decline at higher levels [52]. This non-linear relationship highlights the complexity of PM’s effects on rhinitis and suggests that even low levels of PM exposure may be significant in terms of CRS risk.

The association between PM exposure and rhinitis is further supported by a US study that used two national databases to assess the correlation between exposure to fine particulates and self-reported rhinosinusitis. The study found an increased adjusted hazard ratio in most population groups, indicating a higher risk of rhinosinusitis with increased PM exposure [53]. Another study found that higher levels of PM2.5 were associated with a 1.89-fold increase in the demand for surgery to treat CRS without nasal polyps [54]. This suggests that PM exposure not only increases the risk of developing CRS, but may also exacerbate its severity, leading to a greater need for surgical intervention.

However, it is important to note that the relationship between PM and rhinitis is not consistently observed across all studies. A meta-analysis of six birth cohorts reported no increased risk of AR from exposure to PM2.5 [OR: 1.02 per 2 µg/m^3^ increase in PM2.5 (95% CI: 0.72–1.43)] [55]. Two European longitudinal cohort studies did not find an association between increased exposure to PM2.5 and rhinitis [56]. This discrepancy highlights the need for further research and suggests that the relationship between PM and rhinitis may be influenced by factors such as geographical location, study design, and population characteristics.

### 3.4. Nutritional Supplements and Their Immunomodulatory Role

Nutritional supplements with immunomodulatory properties have garnered significant attention in the context of respiratory health, particularly in relation to air pollution-induced conditions such as AR and CRS. These supplements offer potential protective and therapeutic effects against the inflammatory responses triggered by PM exposure. Table 1 summarizes these supplements and their potential preventive roles in rhinitis.

#### 3.4.1. Kefir Peptides

- The supplement

Kefir, a fermented milk beverage originating from the Caucasus region, has gained recognition for its potential health benefits, including its immunomodulatory properties. It is produced by fermenting milk with kefir grains, a complex symbiotic community of bacteria and yeasts embedded in a polysaccharide matrix called kefiran. Kefir contains various bioactive compounds, including lactic acid bacteria (*Lactobacillus* species), yeasts, kefir exopolysaccharides (KEPS, kefiran), kefir peptides (KPs, KFP-1, KFP-3), short-chain fatty acids, and organic acids, which contribute to its health-promoting effects [57]. The potential of kefir as a therapeutic agent in managing rhinitis associated with PM exposure has been explored due to its ability to modulate immune responses and protect against oxidative stress.

- Immunomodulatory role

The immunomodulatory role of kefir in rhinitis involves several key mechanisms. Kefir can influence the balance between T helper 1 (Th1) and T helper 2 (Th2) cells, which play crucial roles in immune responses. Th2 cells are predominantly involved in allergic responses, including the production of IgE antibodies and the activation of mast cells, leading to the release of inflammatory mediators like histamine. Kefir has been shown to promote a shift towards Th1 responses, thereby suppressing Th2-mediated allergic inflammation in the nasal mucosa [58].

Kefir also exhibits antioxidant properties, which can protect against oxidative stress induced by PM exposure. PM can generate reactive oxygen species (ROS), which can damage cells and tissues and contribute to inflammation. Kefir’s antioxidant components, such as peptides and organic acids, can scavenge ROS and mitigate the adverse effects of PM exposure [59]. Furthermore, kefir can modulate the gut microbiota, promoting a healthy balance of bacteria and influencing immune responses in the lungs [60].

Kefir peptides can also inhibit inflammation caused by the NF-κB pathway [59,62], decrease the expression of downstream inflammatory mediators, and prevent the expression of adhesion proteins (ICAM-1, VCAM-1) so that immunocytes in the circulatory system cannot enter the site of inflammation and damage the tissue [61].

- Animal studies

Animal studies have provided evidence supporting the immunomodulatory role of kefir in PM-induced respiratory disease. Lee et al. (2007) conducted a study on the anti-inflammatory and anti-allergic effects of kefir in a mouse asthma model [58]. They found that oral administration of kefir significantly suppressed airway hyperresponsiveness, a hallmark of asthma. Furthermore, kefir reduced the number of eosinophils, key inflammatory cells involved in allergic responses, in both bronchoalveolar lavage fluid and lung tissue. The study also observed a decrease in the levels of Th2 cytokines, indicating a suppression of the allergic response. Finally, kefir attenuated mucus hypersecretion by goblet cells in the airway, which contributes to airway obstruction in asthma.

In another study, Chen et al. (2019) focused on the effects of kefir peptides on pulmonary inflammation induced by particulate matter (PM4.0) exposure [59]. Utilizing luciferase transgenic mice, which express a light-emitting enzyme in response to the activation of the NF-κB pathway, a key inflammatory signaling pathway, they observed that oral administration of kefir peptides reduced lung inflammation. This was evidenced by decreased luciferase activity, indicating suppression of the NF-κB pathway. Additionally, kefir peptides decreased the levels of pro-inflammatory cytokines and chemokines in both bronchoalveolar lavage fluid and lung tissue. The study also highlighted the antioxidant properties of kefir peptides, as they attenuated oxidative stress in the lungs.

- Clinical studies

While clinical studies specifically investigating the effects of kefir on PM-related rhinitis are limited, some research suggests potential benefits. A study by Dębińska and Sozańska (2022) reviewed the role of fermented foods, including kefir, in asthma and respiratory allergies [88]. The review highlighted the potential of kefir to exert anti-allergic effects through immune modulation and gut microbiota influence.

#### 3.4.2. Lactoferrin

- The supplement

Lactoferrin (LF) is a multifunctional glycoprotein found in various bodily fluids, including milk, tears, saliva, and nasal secretions. It possesses a range of biological activities, including iron-binding, antimicrobial, antioxidant, and immunomodulatory properties [63]. Furthermore, LF shows promise as a safe and natural adjunctive treatment, particularly in pediatrics, where natural substances are widely perceived as beneficial and health-promoting. Its potential role in reducing the need for ongoing prophylaxis and managing allergic disorders is underscored by its anti-inflammatory, immunomodulatory, and antioxidant properties [89]. The potential of LF as a therapeutic agent in managing rhinitis associated with PM exposure has garnered increasing attention due to its ability to modulate immune responses and protect against oxidative stress.

- Immunomodulatory role

The immunomodulatory role of LF in rhinitis is complex and involves several key mechanisms. LF can influence the balance between Th1 and Th2 cells, which play crucial roles in immune responses. Th2 cells are predominantly involved in allergic responses, including the production of IgE antibodies and the activation of mast cells, leading to the release of inflammatory mediators like histamine. LF has been shown to promote a shift towards Th1 responses, thereby suppressing Th2-mediated allergic inflammation in the nasal mucosa [64]. Moreover, LF has been shown to downregulate the production of pro-inflammatory cytokines, such as TNF-α, IL-1β, and IL-6, while upregulating the production of anti-inflammatory cytokines, such as IL-10 [65]. This modulation of cytokine production can help to reduce inflammation and allergic symptoms in the nasal mucosa. LF also exhibits antioxidant properties, which can protect against oxidative stress induced by PM exposure. LF can scavenge ROS and upregulate the expression of antioxidant enzymes, thereby mitigating the adverse effects of PM exposure [66].

- Animal studies

Several animal studies have investigated the immunomodulatory role of LF in rhinitis related to PM exposure. A study by Wang et al. (2013) demonstrated that intranasal administration of recombinant human LF (rhLF) attenuated allergic inflammation in a murine model of allergic rhinitis [67]. The treatment reduced the number of eosinophils and goblet cells in the nasal mucosa, as well as the expression of Th2-related cytokines. Another study investigated the protective effects of LF in a mouse model of ovalbumin-induced asthma. The study found that oral administration of LF reduced airway hyperresponsiveness, pulmonary inflammation, and expression of Th2 cytokines. LF also decreased the secretion of allergen-specific antibodies and influenced the function of dendritic cells [65].

- Clinical studies

Clinical studies evaluating the efficacy of LF supplementation in PM-related rhinitis are limited. However, a study by Passali et al. (2015) showed that a multicomponent device containing LF improved AR symptoms in children [68]. The improvement was evaluated using a VAS (visual analogue scale) and objective measures such as ARR (active anterior rhinomanometry) and MTT (mucociliary transport time).

#### 3.4.3. Vitamin D

- The supplement

Vitamin D is a fat-soluble secosteroid hormone that plays a crucial role in regulating the immune system and maintaining respiratory health. It is primarily synthesized in the skin upon exposure to ultraviolet B (UVB) radiation, and can also be obtained through dietary sources such as fatty fish, egg yolks, and fortified foods [69].

- Immunomodulatory role

The immunomodulatory effects of vitamin D in the context of rhinitis have been extensively studied. Vitamin D has been shown to exert anti-inflammatory properties by modulating the differentiation and function of various immune cells, including T cells, B cells, and dendritic cells [69,70,71]. In the setting of AR, vitamin D has been found to suppress the production of pro-inflammatory cytokines, such as IL-4 and IL-17, while promoting the secretion of the anti-inflammatory cytokine IL-10 [71].

- Animal studies

Animal studies have provided valuable insights into the mechanisms by which vitamin D can mitigate PM-induced inflammation in rhinitis. In a mouse model of AR, supplementation with vitamin D3 was found to attenuate the inflammatory response triggered by exposure to ovalbumin, as evidenced by reduced nasal symptoms, decreased levels of inflammatory cytokines, and improved nasal mucosal integrity [71]. The protective effects of vitamin D were attributed to its ability to downregulate the secretion of IL-4, thereby dampening the pro-inflammatory cascade.

- Clinical studies

Several clinical studies have investigated the correlation between vitamin D and rhinitis. A meta-analysis of eight studies involving 337 chronic rhinosinusitis patients and 179 healthy controls reported a significantly lower level of serum vitamin D in the CRS patients [90]. Another study involving 60 CRS patients reported a significantly lower serum vitamin D concentration among CRS patients with nasal polyposis. Vitamin D deficiency correlated significantly with the severity of CRS and nasal polyp formation [91]. A clinical trial involving 60 patients with chronic rhinosinusitis, nasal polyposis, and low serum Vitamin D levels undergoing endoscopic sinus surgery demonstrated that vitamin D supplementation resulted in a significant reduction in symptom severity and incidence of recurrence and improved endoscopic scores compared to the placebo [72].

Clinical studies on the preventive effects of vitamin D supplementation on PM exposure-related rhinitis are limited. One study investigated the impact of vitamin D levels on the relationship between indoor fine particulate matter (PM2.5) exposure and asthma symptoms in urban children [73]. The research involved 120 children with asthma, monitoring their indoor PM2.5 exposures, vitamin D levels, and asthma symptoms over nine months. The findings revealed that obese children with low vitamin D levels experienced heightened daytime asthma symptoms associated with PM2.5 exposure. Conversely, in high PM2.5 environments, adequate vitamin D levels offered protection against asthma symptoms in obese children. The study concludes that maintaining optimal vitamin D status in children could help mitigate the adverse respiratory effects of indoor air pollution, particularly in obese children, who appear to be more susceptible.

#### 3.4.4. Polyunsaturated Fatty Acids

- The supplement

Polyunsaturated fatty acids (PUFAs) are essential fatty acids that the human body cannot synthesize and must be obtained from dietary sources. They are categorized into omega-3 and omega-6 fatty acids based on the position of the first double bond from the methyl end of the fatty acid chain. Omega-3 fatty acids, primarily found in fatty fish, flaxseed, and chia seeds, include alpha-linolenic acid (ALA), eicosapentaenoic acid (EPA), and docosahexaenoic acid (DHA) [74]. These fatty acids are recognized for their anti-inflammatory and immunomodulatory properties, making them potential candidates for mitigating the adverse effects of particulate matter (PM) exposure, including rhinitis.

- Immunomodulatory role

The immunomodulatory role of omega-3s and other PUFAs in rhinitis is multifaceted and involves several key mechanisms. PUFAs can influence the balance between Th1 and Th2 cells, which play crucial roles in immune responses. Th2 cells are predominantly involved in allergic responses, including the production of IgE antibodies and the activation of mast cells, leading to the release of inflammatory mediators like histamine. Omega-3 fatty acids have been shown to promote a shift towards Th1 responses, thereby suppressing Th2-mediated allergic inflammation in the nasal mucosa [75,76].

Furthermore, PUFAs can modulate the production of lipid mediators, which are bioactive molecules derived from fatty acids that regulate various physiological processes, including inflammation. Omega-3 fatty acids are precursors to anti-inflammatory lipid mediators such as resolvins and protectins, which actively promote the resolution of inflammation and counteract the effects of pro-inflammatory mediators [77]. In the context of rhinitis, these anti-inflammatory lipid mediators can help reduce nasal inflammation, mucus production, and other allergic symptoms.

- Animal studies

Animal studies on the protective role of PUFAs against PM-induced rhinitis are limited. One study investigated the immunomodulatory role of omega-3s and other PUFAs in respiratory pathology related to PM exposure, including rhinitis. Research has shown that dietary supplementation with fish oil, which is rich in EPA and DHA, can attenuate PM2.5-induced lung injury in mice by reducing inflammation and oxidative stress and modulating the gut microbiota [78]. These findings suggest that omega-3 fatty acids may offer protection against the adverse respiratory effects of PM exposure by modulating immune responses and reducing inflammation.

- Clinical studies

Clinical data on the preventive role of PUFAs in PM-related sinusitis are also limited. A randomized, double-blind, placebo-controlled clinical trial is currently underway to investigate the effect of omega-3 fatty acids on Th1/Th2 cell polarization in individuals with high exposure to PM2.5 in the Chengdu subway station [79]. The study aims to evaluate the impact of omega-3 supplementation on various clinical outcomes, including changes in the Th1/Th2 cell polarization index, serum cytokine concentrations, early indicators of atherosclerosis, pulmonary function, and quality of life. The results of this trial are expected to provide valuable evidence regarding the potential benefits of omega-3 fatty acids in mitigating the adverse health effects of PM exposure in humans.

#### 3.4.5. Probiotics

- The supplement

Probiotics are live microorganisms that, when administered in adequate amounts, confer a health benefit on the host. The concept of probiotics dates back to the early 20th century, when Elie Metchnikoff proposed that consuming beneficial bacteria could improve health and longevity [80]. Today, probiotics are widely recognized for their potential to modulate the immune system, improve gut health, and contribute to overall well-being [81]. In the context of rhinitis related to PM exposure, probiotics have emerged as a potential therapeutic strategy due to their immunomodulatory properties and ability to influence the gut–lung axis.

- Immunomodulatory role

The immunomodulatory role of probiotics in rhinitis involves several key mechanisms. Probiotics can influence the balance between Th1 and Th2 cells, which play crucial roles in immune responses. Th2 cells are predominantly involved in allergic responses, including the production of IgE antibodies and the activation of mast cells, leading to the release of inflammatory mediators like histamine. Probiotics have been shown to promote a shift towards Th1 responses, thereby suppressing Th2-mediated allergic inflammation in the nasal mucosa [82].

In addition to modulating the Th1/Th2 balance, probiotics can also enhance the function of regulatory T cells (Tregs), which play a crucial role in maintaining immune tolerance and preventing excessive immune responses. Tregs suppress the activation and proliferation of effector T cells, thereby limiting inflammation and promoting tissue repair. Probiotics have been shown to increase the number and activity of Tregs, which may contribute to their beneficial effects in AR [83]. Furthermore, probiotics can influence the gut–lung axis, a bidirectional communication pathway between the gut and the respiratory system. The gut microbiota play a crucial role in immune system development and function, and alterations in gut microbiota composition have been linked to various respiratory diseases, including asthma and AR. Probiotics can modulate the gut microbiota, promoting a healthy balance of bacteria and influencing immune responses in the lungs. This modulation of the gut–lung axis may contribute to the beneficial effects of probiotics in PM-related rhinitis [84].

- Animal studies

A study by Lee et al. (2023) demonstrated that oral administration of Lactobacillus paracasei ATG-E1 suppressed immune activation and airway inflammatory responses in a PM10D-induced airway inflammation mouse model [85]. The probiotic treatment reduced the neutrophil infiltration and the number of various immune cells in the bronchoalveolar lavage fluid (BALF) and lungs. It also suppressed the expression of pro-inflammatory cytokines and chemokines, and protected against histopathological damage in the lungs.

- Clinical studies

Clinical studies evaluating the efficacy of probiotics in PM-related rhinitis are limited, but emerging evidence suggests potential benefits. A recent systematic review and meta-analysis by Luo et al. (2022) reported that probiotics improved the quality of life, total nasal symptom scores, and ocular symptom scores in patients with AR [86]. The analysis also showed that probiotics influenced the Th1/Th2 ratio, suggesting an immunomodulatory effect. Another study by Anania et al. (2021) investigated prophylactic treatment with a probiotic mixture containing *Bifidobacterium animalis* subsp. lactis BB12 and *Enterococcus faecium* L3 in children with AR [87]. The probiotic mixture was administered three months before the pollen season, and the results showed that the children treated with probiotics experienced less severe symptoms and used less rescue medication compared to the control group.

## 4. Conclusions

This review has explored the relationship between air pollution, particularly PM, and rhinitis in Taiwan, revealing a strong association between PM exposure and increased risk of both allergic and non-allergic rhinitis. The inflammatory mechanisms triggered by PM, including oxidative stress and immune dysregulation, play crucial roles in rhinitis pathogenesis, highlighting the need for effective mitigation strategies.

Our exploration of nutritional supplements as potential preventive measures has shown promise. Kefir peptides, lactoferrin, vitamin D, polyunsaturated fatty acids, and probiotics have demonstrated varying degrees of efficacy in modulating immune responses and reducing inflammation associated with air pollution exposure. However, evidence specific to their efficacy in preventing or managing PM-induced rhinitis in Taiwan, as well as elsewhere, remains limited. Although not all nutritional supplements mentioned above were investigated in Taiwan, we organized research articles from other countries to supplement the limited research in Taiwan and to enrich the potential applications of these nutritional supplements in PM-induced rhinitis. In Table 1, we referred to the research findings from Taiwan and other countries to provide useful insights for PM-induced rhinitis in Taiwan.

Future research should focus on long-term clinical trials to establish the efficacy and safety of these supplements in the Taiwanese population, investigate their specific mechanisms of action, explore potential synergistic effects, and conduct cost-effectiveness analyses. Additionally, studies on personalized nutritional interventions could enhance the effectiveness of these preventive approaches.

While nutritional supplements offer a promising complementary approach, they should not be seen as a substitute for broader environmental policies. Instead, they should be part of a comprehensive strategy including stringent air quality regulations and public education on air pollution mitigation.

## Figures and Tables

**Table 1:** Nutritional supplements with possible preventive roles against particulate matter (PM)-induced rhinitis.

**Supplement**	**Definition**	**Immunomodulatory Mechanism of Action**	**Possible Role in Rhinitis and Other Respiratory Conditions**
Kefir peptides	Bioactive compounds derived from fermented milk beverages [57]	- Shifts immune response from Th2 to Th1 [58] - Exhibits antioxidant properties [59] - Modulates gut microbiota [60]. - Inhibits NF-κB pathway [59,61,62]	- Suppresses airway hyperresponsiveness [58] - Attenuates mucus hypersecretion [58]
Lactoferrin	Multifunctional glycoprotein found in bodily fluids [63]	- Promotes shift from Th2 to Th1 responses [64] - Downregulates pro-inflammatory cytokines [65] - Upregulates anti-inflammatory cytokines [65] - Exhibits antioxidant properties [66]	- Attenuates allergic inflammation in nasal mucosa [67] - Improves AR symptoms in children [68]
Vitamin D	Fat-soluble secosteroid hormone [69]	- Modulates differentiation and function of immune cells [69,70,71] - Suppresses pro-inflammatory cytokines [71] - Promotes anti-inflammatory cytokine secretion [71]	- Attenuates inflammatory response in AR [71] - Improves nasal mucosal integrity [71] - Reduces symptom severity in CRS with nasal polyposis [72] - May protect against asthma symptoms in obese children exposed to PM2.5 [73]
Polyunsaturated fatty acids (PUFAs)	Essential fatty acids obtained from dietary sources [74]	- Promotes shift from Th2 to Th1 responses [75,76] - Modulates production of anti-inflammatory lipid mediators [77]	- Reduces nasal inflammation and mucus production [75,76] - Attenuates PM2.5-induced lung injury (in animal studies) [78] - May modulate immune responses and reduce inflammation in PM-exposed individuals [79]
Probiotics	Live microorganisms that confer health benefits when administered adequately [80,81]	- Promotes shift from Th2 to Th1 responses [82] - Enhances function of regulatory T cells [83] - Modulates gut–lung axis [84]	- Suppresses immune activation and airway inflammatory responses in PM-induced inflammation [85] - Improves quality of life and nasal symptom scores in AR patients [86] - Reduces symptom severity and rescue medication use in children with AR [87]

AR: allergic rhinitis; CRS: chronic rhinosinusitis; PM: particulate matter; Th1: T helper 1 cells; Th2: T helper 2 cells.

## Data Availability

All data supporting this review are incorporated within the article.
